# A large-scale image dataset of wood surface defects for automated vision-based quality control processes

**DOI:** 10.12688/f1000research.52903.1

**Published:** 2021-07-16

**Authors:** Pavel Kodytek, Alexandra Bodzas, Petr Bilik

**Affiliations:** 1 Department of Cybernetics and Biomedical Engineering, VSB-Technical University of Ostrava, Ostrava, 70800, Czech Republic

**Keywords:** wood surface defects, high resolution dataset, wood industry, wood processing, wood quality control process, wood defects dataset

## Abstract

The wood industry is facing many challenges. The high variability of raw material and the complexity of manufacturing processes results in a wide range of visible structure defects, which have to be controlled by trained specialists. These manual processes are not only tedious and biased, but also less effective. To overcome the drawbacks of the manual quality control processes, several automated vision-based systems have been proposed. Even though some conducted studies achieved a higher recognition rate than trained experts, researchers have to deal with a lack of large-scale databases and authentic data in this field. To address this issue, we performed a data acquisition experiment set in the industrial environment, where we were able to acquire an extensive set of authentic data from a production line. For this purpose, we designed and implemented a complex technical solution suitable for high-speed acquisition during harsh manufacturing conditions. In this data note, we present a large-scale dataset of high-resolution sawn timber surface images containing more than 43 000 labelled surface defects and covering 10 types of the most common wood defects. Moreover, with each image record, we provide two types of labels allowing researchers to perform semantic segmentation, as well as defect classification, and localization.

## Introduction

In the wood industry, each step of the manufacturing process affects material utilization and cost efficiency. ^1^ The heterogeneity of wood material with the complexity of these manufacturing processes may result in various defects, which not only degrade the mechanical properties of the wood such as the strength and stiffness but also reduce its aesthetic value. ^2^ These mechanical and aesthetical defects have furthermore a large impact on the commercial value of the wood and can diminish the utilization of such materials for further processing. There are many various types of defects arising from many different causes. The major wood defects include knots, fungal damage, cracks, warping, slanting, wormholes, and pitch defects. The seriousness of a defect, and therefore the grade and the cost of the material, is primarily determined by four criteria, including the size, location, type of the defect, and the purpose for which the wooden product will be used. ^3, 4^

Even though the automation in this industrial sector is growing, many market leader companies still utilize trained domain experts to detect undesirable features and to perform quality grading. ^5^ Besides the fact that the manual examination is tedious and biased, it was found that domain experts are not able to check large production volumes. Moreover, the study conducted by Urbonas *et al*. ^6^ stated that due to factors such as eye fatigue or distraction, manual inspection rarely achieves 70 % reliability. To overcome the drawbacks of the manual examination, researchers try to develop automated systems, which are accurate and won't slow down the manufacturing process. According to the repeatability and quality of the inspection, the study performed by Lycken ^7^ has already proved that automatic systems slightly outperformed human graders. Most of these systems were based on conventional image processing techniques in combination with supervised learning algorithms, however, over the last decade deep learning has achieved remarkable success in the forestry and wood products industry. ^8^ Although researchers in the field were able to achieve satisfying results with the average recognition rate above 90 %, ^9^ most of the authors worked with small-scale image datasets obtained in laboratory conditions by using self-developed vision system setups. Performing experiments in such conditions usually entails the disadvantage of a limited number of available products. In most of the studies, ^2, 6, 10, 11^ researchers compensate for the lack of real products by using data augmentation techniques, which can expand the dataset up to 10 times its original size. From one point of view, data augmentation is considered to be an excellent tool to generalize the classification model and therefore prevent overfitting. ^12^ Nonetheless, it cannot ensure that the variability of the observed phenomenon will be sufficiently captured, especially in cases where the variability might be limitless.

In order to address the lack of extensive databases in the field, we performed an experiment with the goal to acquire a large-scale dataset of timber surface defects. Unlike other conducted studies, our experiment was placed in an industrial environment during real production, which allowed us to acquire a large amount of authentic data from the production line. To face the challenges arising from the manufacturing process, such as the high speed of the conveyor belt and heavy vibrations, we designed hardware as well as a software solution, which enabled acquisition of high-resolution images at the acquisition rate of 66 kHz. In this experiment, we acquire 20 276 original data samples of sawn timber surface, from which 1 992 images were without any surface defects, and 18 284 images captured one or more defects covering overall 10 types of common wood surface defects. The most frequent defects include live knots and dead knots, with an overall occurrence in the dataset of 58.8 % and 41.2 %, respectively. Furthermore, to provide more valuable information in this data descriptor, all dataset samples were complemented with two types of labels: a semantic label map for the semantic segmentation and a bounding box label.

## Methods

Due to the industrial environment where the experiment was set, the most challenging part of this work was the dataset acquisition. Performing data acquisition in such an environment entailed several negative factors. One of those factors was that the sawmill production line utilized for this experiment is used for more than 300 days per year, with minimal pauses, which maximizes the manufacturer's profits. Also, we had to deal with the high speed of the sawmill conveyor belt, which reached a value of 9.6 m s ^−1^ at the place of the acquisition. This high speed of the conveyor causes constant heavy vibrations that in some peaks may result in fluctuations that are even centimetres in length. The main goal of the technical solution was therefore to create a robust and at the same time portable construction, which can be easily implemented in the sawmill environment.

### Acquisition equipment

**Figure 1.**
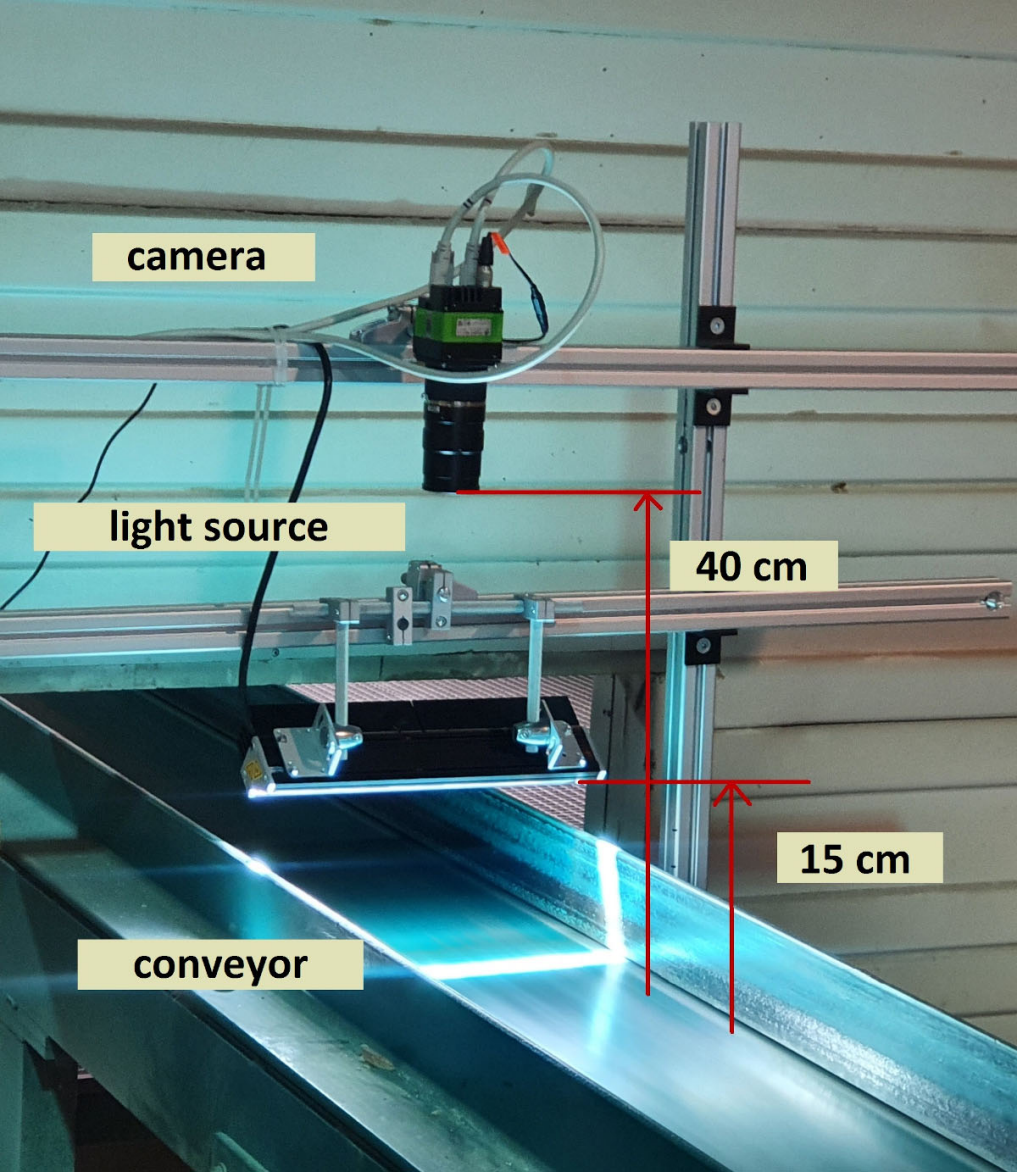
The mechanical construction, including the mounted camera and light source. The distance between the line scan camera and the light source from the conveyor belt is 40 and 15 centimetres, respectively.

To overcome the limitation of this environment, we developed a mechanical construction for carrying the camera and the light source. The final construction assembled from ITEM aluminium profiles was at the place of the acquisition fixed to the production line construction and the floor, which helped to avoid the acquisition of blurry images. Although this solution didn’t deal directly with heavy vibrations, it ensured the harmonization of the conveyor vibrations with the mounted camera. The final mechanical solution implemented in the sawmill environment is demonstrated in Figure 1.

In order to obtain high-quality images at a speed of 9.6 m s ^−1^, a trilinear line scan camera SW-4000TL-PMCL manufactured by JAI was chosen. This camera was able to acquire 3 × 4096 pixels per line at the speed of 66 kHz. The required speed of the image acquisition was achieved by connecting the camera interface to a high-performance Camera Link frame grabber with the transfer speed parameter set to 10 tap mode. For this application, we selected the Silicon Software microEnable five marathon VCLx frame grabber with a PCIe interface that allows on-board high-speed data processing and high data throughput up to 1800 MB s ^−1^. The required field of view, which obtains a part of the sawn timber piece, with a width of 15 cm and the full length of 500 cm was achieved by using the Kowa LM50LF line scan camera lens. The selected camera, together with a 50 mm focal length lens placed at a distance of 40 cm from the measured object, led to a horizontal resolution of 16.66 pixels per millimetre. The vertical resolution *R _v_* of the image was computed before the experiment by the following formula.

$$R_{v}=\frac{1}{\frac{\frac{v_{w}}{60}*L}{v_{c}}}$$ (1)

where *v _w_* is the velocity of the conveyor, *L* is the number of lines per image, and *v _c_* is the line rate of the camera. The resulting vertical resolution of 6.67 pixels per millimeter was afterward experimentally verified during the acquisition process.

Since the shutter of the camera was set to 3 μs to ensure the high-speed image acquisition, we had to use a powerful light source, which would sufficiently illuminate the desired field of view. For this purpose, we selected one of the most powerful light sources on the market, a linear LED light Corona II by Chromasens with the ability to provide a light intensity of 3.5 million lux. To achieve the best possible images, a white spectrum of the light was utilized.

### Data acquisition

Instead of saving every single line during the acquisition process, we captured a block of 1024 lines, which resulted in an image resolution of 1024×4096. Such a high-resolution color image takes up approximately 12 MB of disk space. The used sampling frequency of 66 kHz with the total number of captured pixels resulted in a data transfer speed of 773 MB s ^−1^, which means that we were able to capture 66.4 images per second. Even though we used a very powerful computer, we found the process of saving this amount of data at such a high speed quite challenging. To overcome this challenging task, we had to separate image acquisition and image saving into two separate processes. While the acquisition process consisted of capturing a set of 84 images with a subsequent saving into the PC's RAM, the only task of the saving process was the transfer of the images from the computer RAM to the local hard disk drive. For this experiment, we employed two external 1 TB hard drives. To save CPU time during the acquisition and saving process, no online processing was performed.

Because transferring such a large amount of data between different software have a negative impact on CPU utilization and would decrease the frame rate, we used optimized frame grabber software, microDisplay X (runtime version 5.7) from Silicon Software. ^13^ To use this software in an automated way, we developed an automatic clicker with a feedback loop based on the captured computer screen. In simple terms, the software reads the desired information from the screen and based on the information decides whether the acquisition or saving process is already completed. Additionally, it automatically assigns an incrementing filename to each captured image. This was mainly realized by using Windows library user32.dll, which allows the control of various aspects of mouse motion and button clicking. Since the saving process (loop) is almost 10 times slower than the acquisition process, the acquisition loop had to be temporarily stopped in each cycle. Despite the fact that this causes loss of the data continuity, it does not affect the study validity and reliability. We assumed that the acquisition process with the other support subroutines takes approximately 1.4 s while the saving process lasts 7.5 s. To maintain a predictable acquisition speed, including software delays, we introduced synchronization, which started a new cycle every 9 s.

### Data processing

During four hours of acquisition, 60 480 images were acquired overall. Due to the limited third-party software functionality, the acquisition process had to be performed in a continuous mode, without any triggering option. This resulted in a large number of images of an empty conveyor or partly captured wood surface. To filter these meaningless data from the dataset, an offline histogram-based algorithm was created. The basic idea behind this algorithm is the sum calculation of the image green color space histogram. This sum value of the histogram can be in the next step divided by any number from the range of 5 to 10 (values in the range were deduced from the size of the images). The last step of the algorithm is based on a simple threshold, where all images with a resulting value of less than 10 were removed. Using this value of threshold ensured that only images that contained in the horizontal direction at least 40 % of the wood surface were kept. Since this filtration approach proved 100 % reliable in successfully filtering images with no wooden surface on 1500 randomly selected and manually sorted samples, we applied this filtering algorithm on the whole dataset. The filtering process reduced the dataset to a final number of 20 275 images.

**Figure 2.**
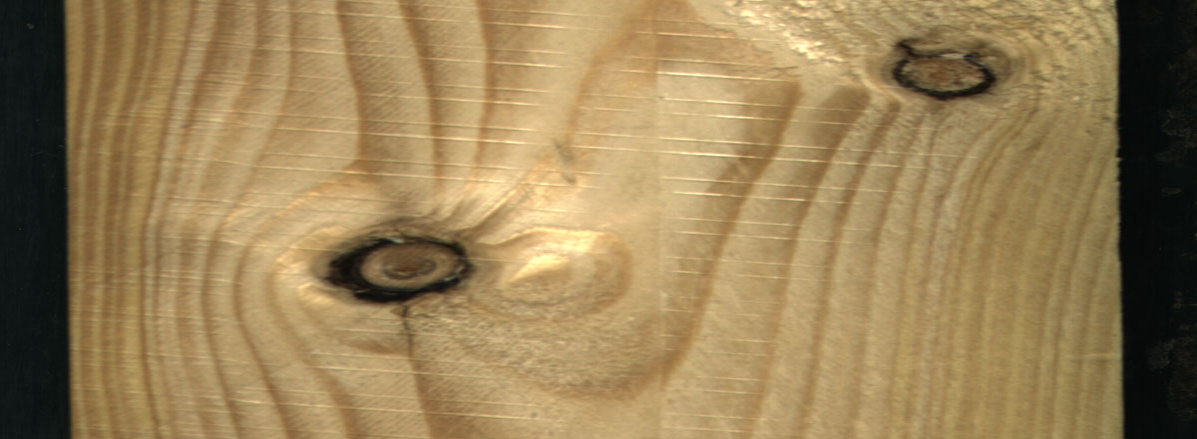
A dataset example of a sawn timber surface with dead knots.

Additionally, besides the filtration, we performed image cropping to remove the undesirable background from the images. This operation not only reduced the file size but also decreased the potential computation time for future use. To automatically crop each image in the dataset without any relevant data loss, we employed a simple straight-line edge detection technique in a vertical direction. Basically, the main principle of the algorithm is finding as many raising edge points in the desired direction as are needed to construct a line. The cropping operation was then performed on the image bounding box derived from the following formula.

$$\mathrm{BB}\left(x_{1},y_{1},x_{2},y_{2}\right)=\left(\frac{L_{x_{1}+}L_{x_{2}}}{2}-150,L_{y_{1}},\frac{L_{x_{1}+}L_{x_{2}}}{2}+2650,L_{y_{2}}\right)$$ (2)

where $\mathrm{BB}\left(x_{1},y_{1},x_{2},y_{2}\right)$ is the cropped bounding box, and $L_{x_{1}y_{2}x_{1}y_{2}}$ stands for the image coordinates of the detected straight edge. Cropping the image, changed the image resolution to 2800 × 1024, and reduced the overall dataset size by almost 80 GB. An example of the image after the image crop operation is demonstrated in Figure 2.

### Ground truth labelling

The dataset annotation in this study was performed manually by a trained person. To accelerate this time-consuming process, we developed a customizable annotation tool. In comparison with other annotation tools available on the market, which didn’t fulfil our requirements, we created a universal application with the ability to manage bounding box labels, as well as labels for the semantic segmentation at the same time. ^14^

For every single image, we created a BMP file representing a semantic map of the labeled defects. During the labeling process, the user manually painted zones in a displayed image, where each zone drawn with a selected color represents a specific defect. Each drawn zone was then automatically bounded with a zone of the particular label and a bounding rectangle. From the created zones, the tool automatically generated coordinates (left, top, right, bottom respectively) in the form of percent divided by 100, where a certain defect is located. For each processed image from the dataset, the annotation tool therefore created a text file including labels and bounding box coordinates and a semantic segmentation map with the configured color labels.

## Data records

The dataset containing the data acquired in this experiment is publicly available. ^15^ The dataset includes 1 992 images of sawn timbers without any defects and 18 283 timber images with one or more surface defects. On average, there are 2.2 defects per image, while only 6.7 % of images contain more than three defects. The highest occurrence of defects, which was captured during the experiment, was 16 defects per image. In this dataset, we present altogether 10 types of wood surface defects, including several types of knots, cracks, blue stains, resins, or marrows. An overall overview of all available wood surface defects with a number of occurrences is summarized in Table 1.

**Table 1.** Wood surface defects included in the database with the number of particular occurrences and an overall occurrence within the dataset.

Defect type	Number of occurrences	Number of images with the defect	Overall occurrence in the dataset [%]
Live knot	21 224	11 912	58.8
Dead knot	11 985	8 350	41.2
Knot with crack	2 276	1 835	9.1
Crack	2 169	1 578	7.8
Resin	3 455	2 624	12.9
Marrow	1 181	1 060	5.2
Quartzity	1 075	847	4,2
Knot missing	503	478	2.4
Blue stain	96	77	0.4
Overgrown	10	6	0.03

Each colour image with a resolution of 2800×1024 is provided in a BMP format in 10 separated zip folders labelled as *Images*. ^15^ Additionally, we provide two types of annotations, semantic label maps, and bounding box labels. Both labels are provided in separate zip folders. The bounding box labels are located in a folder *Bounding_Boxes* and named as *imagenumber_anno.txt*, where the *image number* corresponds to the name of the original image in the dataset. Each original image has therefore one assigned text file, which can have multiple label records for each defect in the image. All bounding box labels have the following structure, where the first record represents the object label, and the subsequent values correspond to left, top, bottom, and right absolute positions of the defect in the image divided by 100.

$$\begin{array}{ccccc} \text{Knot\_OK} & 0,421786 & 0,819336 & 0,571429 & 1,000000 \end{array}$$

**Figure 3.**
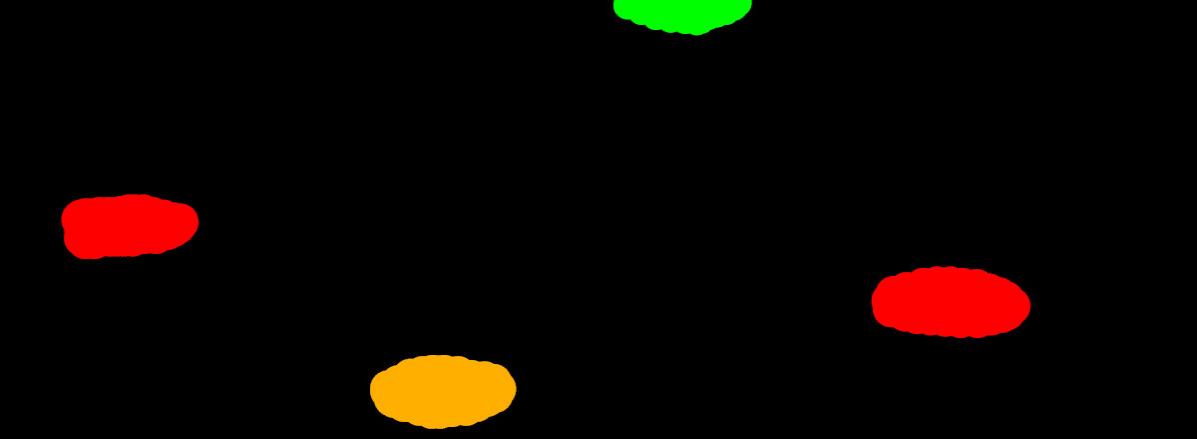
Example of a semantic segmentation label. The red label represents dead knots, the green label stands for live knots, and the dark yellow represents knots with cracks.

Semantic label maps, used for semantic segmentation, are located in a folder, *Semantic Maps.* For each image in the dataset there exists just one semantic map in a BMP format with the label name in the form of *imagenumber_segm.bmp*, where the image number represents the corresponding name of the original image. In comparison to bounding box labels, each pixel of the semantic map image has its label, which is determined by a specified colour (see Figure 3).

To see the exact label specification for the provided wood surface defect dataset, refer to *Semantic Map Specification* text file, ^15^ or Table 2.

**Table 2.** Annotation colour specification for the provided dataset with hexadecimal colour codes.

Defect type	Colour	HEX colour code
Live knot	Green	00FF00
Dead knot	Red	FF0000
Knot with crack	Dark Yellow	FFAF00
Crack	Pink	FF0064
Resin	Magenta	FF00FF
Marrow	Blue	0000FF
Quartzity	Purple	640064
Knot missing	Orange	FF6400
Blue stain	Cyan	10FFFF
Overgrown	Dark Green	004000

## Technical validation

The technical validation of the dataset was conducted by assessing the quality of the assigned labels by employing deep learning-based classification. For this purpose, we utilized a standard state-of-the-art Convolution neural network detector based on the ResNet-50 model. ^16^ The selected neural network architecture was modified by adding Batch Normalization and ReLu layers after each convolution layer. The input layer of the network, and therefore all dataset images were downsampled to 1024×357. To train the neural network, we employed a transfer learning paradigm using pre-trained weights from the COCO dataset. ^17^ Moreover, we performed data augmentation, including horizontal, vertical flip, translation and scaling, and divided the dataset into training and testing set in a conventional ratio of 40/60. To increase the detection of the labelled defects by the ResNet-50 model, several parameters were additionally modified on the basis of the trial-and-error process. These included sizes, strides, ratios and scales (see Table 3).

**Table 3.** A detailed specification of the modified neural network parameters.

Parameter	Values
Sizes	[32, 64, 128, 256, 512]
Strides	[8, 16, 32, 64, 12]
Ratios	[0.3, 0.55, 1, 2, 3.5]
Scales	[0.6, 0.8, 1]

At the beginning of the training, the first four layers of the network were frozen. After freezing the layers, the neural network was tuned by unfreezing the layers in a reverse order except for the Batch Normalization layer. The whole neural network was then finally fine-tuned at a low training speed. The overall number of epochs during the training was 30, while the training speed ranged between 10 ^-4^ at the beginning and 10 ^-6^ at the end of the training.

The trained ResNet-50 model resulted in an accuracy of 81 %. Since the neural network output a large number of false positives, the dataset was re-evaluated by a trained person who didn’t participate in the primary dataset labelling process.

## Data availability

### Underlying data

Zenodo: Underlying data for A large-scale image dataset of wood surface defects for automated vision-based quality control processes. ‘Deep Learning and Machine Vision based approaches for automated wood defect detection and quality control’. http://doi.org/10.5281/zenodo.4694695. ^15^

This project contains the following underlying data:

• Bounding boxes
• Images 1–10
• Semantic map specification
• Semantic maps

Data are available under the terms of the Creative Commons Attribution 4.0 International Public License (CC-BY 4.0).

## Software availability

Zenodo: Software for labeling wood surface defects and managing images. ‘Supporting tools for managing and labeling raw wood defect images’. http://doi.org/10.5281/zenodo.4904736. ^14^

This project contains the following underlying data:

Labeler tool:

SubVI

• Labeler_software.vi
• Readme.txt
• Labeler.ini

Support Utils:

• Cutter.vi
• Sorter.vi

Data are available under the terms of the Creative Commons Attribution 4.0 International Public License (CC-BY 4.0).
