# Supplementary material for: A large-scale image dataset of wood surface defects for automated vision-based quality control processes
Source: F1000Res. 2022 Jun 27;10:581. Originally published 2021 Jul 16. [Version 2] doi: 10.12688/f1000research.52903.2 (PMC9277195; doi:10.12688/f1000research.52903.2)
Supplement: Peer review history

# Peer review: Reviewer response for version 2

**DOI:** 10.5256/f1000research.135431.r142447

**Published:** 2022-07-12

**Authors:** Sri Rahayu

**Affiliations:** 1 Universitas Nusa Mandiri, East Jakarta, Indonesia

The author has fulfilled the suggestions given.

 A minor suggestion: provide some examples of currently available wood defect image datasets from previous researchers, as well as to make more references. Since the paper also produces a wood defect image dataset as well, I think it is better for the author to acknowledge other work to broaden the audience's view that other similar datasets are also available. If I had to mention examples of currently available wood defect image datasets from previous researchers, it is Riana *et al*., 2021 ^1^.

Are sufficient details of methods and materials provided to allow replication by others?

Partly

Is the rationale for creating the dataset(s) clearly described?

Yes

Are the datasets clearly presented in a useable and accessible format?

Partly

Are the protocols appropriate and is the work technically sound?

Yes

Reviewer Expertise:

Single-cell technologies

I confirm that I have read this submission and believe that I have an appropriate level of expertise to confirm that it is of an acceptable scientific standard.

## References

[1] : Comparison of segmentation and identification of swietenia mahagoni wood defects with augmentation images. *Heliyon* .2021;7(6) : 10.1016/j.heliyon.2021.e07417 e07417 10.1016/j.heliyon.2021.e07417 34307930PMC8258648

# Peer review: Reviewer response for version 2

**DOI:** 10.5256/f1000research.135431.r142448

**Published:** 2022-07-08

**Authors:** Mariusz Pelc

**Affiliations:** 1 Faculty of Electrical Engineering, Automatic Control and Informatics, Opole University of Technology, Opole, Poland; 2 School of Mathematical and Computing Sciences, University of Greenwich, London, UK

I have read the revised version of the paper and I am happy to say that the paper has now reached the appropriate standard for indexing. Authors have made changes to the key elements (including use of English).

Are sufficient details of methods and materials provided to allow replication by others?

Yes

Is the rationale for creating the dataset(s) clearly described?

Yes

Are the datasets clearly presented in a useable and accessible format?

Yes

Are the protocols appropriate and is the work technically sound?

Yes

Reviewer Expertise:

Computer science, data / signal processing, automation and robotics, bio-medical engineering, expert sytsems.

I confirm that I have read this submission and believe that I have an appropriate level of expertise to confirm that it is of an acceptable scientific standard.

# Peer review: Reviewer response for version 1

**DOI:** 10.5256/f1000research.56234.r136809

**Published:** 2022-05-26

**Authors:** Sri Rahayu

**Affiliations:** 1 Universitas Nusa Mandiri, East Jakarta, Indonesia

Wood defect datasets are still rare, so this research is very helpful for the wood industry and researchers interested in this field. The authors explain in detail the reasons for building this database with clear data acquisition and collection techniques.

 Some of the methods used to construct semantic images and image testing may be replicated in others. The image acquisition technique used will be of great value if you mention the acquisition technique in other similar studies.

 It would be even better if the authors provide examples of images for each image class and also presents the stages of the research in the form of a chart

Are sufficient details of methods and materials provided to allow replication by others?

Partly

Is the rationale for creating the dataset(s) clearly described?

Yes

Are the datasets clearly presented in a useable and accessible format?

Partly

Are the protocols appropriate and is the work technically sound?

Yes

Reviewer Expertise:

Single-cell technologies

I confirm that I have read this submission and believe that I have an appropriate level of expertise to confirm that it is of an acceptable scientific standard, however I have significant reservations, as outlined above.

# Response

**Published:** 2022-06-22

**Authors:** Alexandra Bodzas

**Affiliations:** VSB - Technical University of Ostrava, Czech Republic

The point-by-point responses to comments:

 1. We complemented the Introduction section with a paragraph describing wood defects acquisition techniques used in other studies (Paragraph 3).

 2. We complemented the paper with a diagram presenting the particular stages of the dataset acquisition (Figure 1) and a figure containing image examples for each class (Figure 4).

# Peer review: Reviewer response for version 1

**DOI:** 10.5256/f1000research.56234.r100946

**Published:** 2021-11-25

**Authors:** Mariusz Pelc

**Affiliations:** 1 Faculty of Electrical Engineering, Automatic Control and Informatics, Opole University of Technology, Opole, Poland; 2 School of Mathematical and Computing Sciences, University of Greenwich, London, UK

This paper deals with a relevant problem which is detection of wood surface defects.

 For the purpose of the research, the authors have come up with a coherent methodology allowing them to acquire all required data, then mapping and detecting defects. From an algorithmic viewpoint it all makes sense, besides, the whole methodology/algorithm validation has also been performed.

 So, the paper ticks pretty much all the boxes (relevance, novelty, etc.) and as such it qualifies for publication.

 However, the paper requires some substantial changes in the following areas:

1. There is no related work section which makes it really difficult to understand the authors' contribution to the field. I would recommend adding such a section (even if it is brief) where similar solutions would be discussed and confronted with what the authors are proposing in this paper.
2. Every single paper should include a conclusion section allowing all readers to understand key findings of the research. This paper is lacking a conclusion section which is quite an omission.
3. Some tables (e.g. Table 1) should be re-done as their versions included in the paper are hardly readable. Usually one look at a table provides a lot of information about the results whilst in this paper this is not the case. I would suggest the authors re-format all tables to make all the dates gathered in the table easy to see and understand.
4. The "Software availability" section should be rewritten. I would suggest the authors make this section easily comprehensible via adding some more description of the software tools used and maybe outline some key feature(s) of the software. Also, based on the section contents, the section title better reflecting this would be e.g. "Supporting software tools" where first paragraph should say that in this research the following software was used (then outline the software and how it was used).
5. Referencing - I only want to make sure that the authors have used the proper referencing style since the most frequently used are either Harvard or IEEE, whilst the authors have used a foot-note like referencing style.
6. The whole paper is written in maybe not error-free but still quite coherent and comprehensible English. But I would still recommend at least one more proof reading to make sure that there are no obvious mistakes left in the text.

Based on the above consideration I would recommend accepting the paper for indexing after revision.

Are sufficient details of methods and materials provided to allow replication by others?

Yes

Is the rationale for creating the dataset(s) clearly described?

Yes

Are the datasets clearly presented in a useable and accessible format?

Yes

Are the protocols appropriate and is the work technically sound?

Yes

Reviewer Expertise:

Computer science, data / signal processing, automation and robotics, bio-medical engineering, expert sytsems.

I confirm that I have read this submission and believe that I have an appropriate level of expertise to confirm that it is of an acceptable scientific standard, however I have significant reservations, as outlined above.

# Response

**Published:** 2022-06-22

**Authors:** Alexandra Bodzas

**Affiliations:** VSB - Technical University of Ostrava, Czech Republic

The point-by-point responses to comments:

 1. Our paper was written in accordance with the journal guidelines for a Data Note article, which slightly differs from an original research article. Concerning the Data Note article, there is no related work section included in the article structure. However, to fulfill your requirements, we complemented the introduction section with a paragraph where we discussed similar solutions. The importance of this research is then explained in subsequent paragraphs.

 2. The conclusion section is omitted intentionally again since we followed the data note article guidelines.

 3. The tables in the article cannot be re-formatted since the article had been formatted by the editorial team before the publication. Table formatting is within the scope of an editorial team that formats the table according to the journal standards. Since the article is an online article, the tables are accessible and visible in full size after clicking on them.

 4. The original Software availability section was rewritten before the publication to fulfill the editorial team's requirements. However, we complemented this section by adding some descriptions of the software. The title of this section cannot be changed since it follows the guidelines for a data note article and journal standards.

 5. We unified the reference styles within the references. All references in a reference list are according to the Harvard referencing style. The in-text citation on the other hand are according to the journal standards, and the footnotes were added by the editorial team during the typesetting process

 6. The paper was proofread and edited. The obvious mistakes were corrected.
